# Building pathway clusters from Random Forests classification using class votes

**DOI:** 10.1186/1471-2105-9-87

**Published:** 2008-02-06

**Authors:** Herbert Pang, Hongyu Zhao

**Affiliations:** 1 Division of Biostatistics, Department of Epidemiology and Public Health, Yale University School of Medicine, New Haven, CT 06520, USA; 2 Department of Genetics, Yale University School of Medicine, New Haven, CT 06520, USA

## Abstract

**Background:**

Recent years have seen the development of various pathway-based methods for the analysis of microarray gene expression data. These approaches have the potential to bring biological insights into microarray studies. A variety of methods have been proposed to construct networks using gene expression data. Because individual pathways do not act in isolation, it is important to understand how different pathways coordinate to perform cellular functions. However, there are no published methods describing how to build pathway clusters that are closely related to traits of interest.

**Results:**

We propose to build pathway clusters from pathway-based classification methods. The proposed methods allow researchers to identify clusters of pathways sharing similar functions. These pathways may or may not share genes. As an illustration, our approach is applied to three human breast cancer microarray data sets. We found that our methods yielded consistent and interpretable results for these three data sets. We further investigated one of the pathway clusters found using PubMatrix. We found that informative genes in the pathway clusters do have more publications with keywords, like estrogen receptor, compared with informative genes in other top pathways. In addition, using the shortest path analysis in GeneGo's MetaCore and Human Protein Reference Database, we were able to identify the links which connect the pathways without shared genes within the pathway cluster.

**Conclusion:**

Our proposed pathway clustering methods allow bioinformaticians and biologists to investigate how informative genes within pathways are related to each other and understand possible crosstalk between pathways in a cluster. Therefore, building pathway clusters may lead to a better understanding of molecular mechanisms affecting a trait of interest, and help generate further biological hypotheses from gene expression data.

## Background

The increasing use of high-throughput microarray technologies in biological and biomedical research has motivated many novel statistical and computational approaches to analyze such data. They can be applied to (1) identify differentially expressed genes, (2) discover subclasses through clustering, and (3) classify subjects into known classes. Although most of these methods either examine one gene at a time, i.e. single-gene based, or all the genes at the same time, a number of methods investigate a set of genes at a time, where the gene-set information can come from various external databases, such as KEGG [1], BioCarta [2] and GenMapp [3]. These curated gene-sets or pathways from biological experiments often serve a particular cellular or physiological function. These gene-set based (or pathway-based) methods include Gene Set Enrichment Analysis (GSEA) [4], Random Forests [5], Hotelling's T^2 ^[6], and Significance Analysis of Microarray to gene-set analyses (SAM-GS) [7]. Although it is unlikely that one particular method will be superior to others for all the data sets, these methods seem to be able to generate biologically meaningful results for different data sets. In addition, pathway-based tests can identify more subtle changes in expression than single gene based tests [8]. Furthermore, pathway-based methods can generate biological hypotheses more effectively based on prior knowledge. These hypotheses may be readily tested using complementary approaches, e.g. proteomics and metabolomics analyses.

It is well known that different pathways do not work in isolation. In fact, each pathway is part of an overall biological network. Therefore, it is natural to ask how different pathways, or gene-sets, coordinate their activities. In the context of using gene expression data to predict a trait of interest, e.g. cancer, some pathways may function in a coherent fashion whereas others may have independent functions or effects on phenotypes. Despite the importance of this topic, there is scant literature on relating different pathways. In this paper, we propose to cluster pathways that have similar effects on the phenotype of interest. Our approach is built on our previous proposal of adopting the Random Forests approach for pathway analysis [5]. The Random Forests approach has been found to perform very well among a number of machine learning methods in pathway-based classification. To extend the Random Forests approach for pathway cluster analysis, we use class votes from Random Forests to build pathway clusters related to phenotype of interest. As detailed below in the Methods section, class votes can provide a measure of the similarity between two subjects' gene expression profiles for a given pathway. This measure can then be used to define similarities, or distances, between pathways. Based on these inferred pathway distances, we then use the Tight Clustering [9] approach to identify pathway clusters. The identification of such clusters may provide useful information for biologists to generate hypotheses on the underlying disease mechanisms. Pathway clusters may also help identify novel biomarkers for screening or serving as drug targets for combination therapy.

The rest of the paper is organized as follows. The detailed methodology is discussed in the Methods section. In the Results section, we demonstrate the usefulness of this approach through the application of our methods to three different breast cancer microarray data sets to uncover pathway clusters that are involved in estrogen receptor (ER) status classification. We conclude the paper in the Discussion and Conclusions sections.

## Methods

We first briefly review the Random Forests approach for pathway analysis [5]. Random Forests constructs many classification trees and thus the name 'forest'. For each pathway, the input data for Random Forest would be a gene expression matrix of the genes belonging to the pathway by the number of subjects in the data set. Every tree in a Random Forests is built using a deterministic algorithm and the trees are different from the ordinary tree algorithms (e.g. CART) owing to two factors. First, at each node, a best split is chosen from a random subset of the predictors rather than all of them. Second, every tree is built using a bootstrap sample of the original observations. A subject is put down a tree for classification using the input vector of gene expression for genes within a particular pathway. The tree gives a classification and decides which class this subject belongs to. In the end, the forests choose the class that gives the majority votes for each subject. The out-of-bag (OOB) data, approximately one-third of the observations, are then used to estimate the prediction accuracy. Small classification error based on genes in a given pathway would indicate the pathway as potentially interesting [5].

We can build for each pathway a Random Forest to predict an individual's phenotype based on his/her gene expression levels within this pathway. To define whether two pathways have similar effects on an individual's phenotype, we can use the pathway prediction results to define their similarities. For example, if two pathways always give the same phenotype prediction based on gene expression data in these pathways, we infer that these two pathways have similar functions. To realize this idea, we use an output from Random Forests, class votes, to define pathway distances that can be used to build pathway clusters.

### Class Votes

To define class votes, for each study subject, the proportion of votes for a specific class is recorded based on the prediction results from individual trees in the Random Forest. Therefore, every pathway defines a class vote matrix of length n by k, where n is the number of samples in the study and k is the number of classes. In case of only two classes, we can use the votes of one class to represent the confidence of each individual belonging to that particular class. For example, for subject A, if we have 0.15 for class 1 and 0.85 for class 2, that means subject A has been voted to be class 2 85% of the time. Therefore, two pathways can be thought to have similar effects on the phenotype if the class vote matrices/vectors from these two pathways are similar.

### Building Pathway Clusters

Based on class votes, we propose to use Tight Clustering to infer pathway clusters.

Tight Clustering is a robust method using re-sampling for clustering and pattern recognition [9]. It finds tight and stable clusters in a sequential manner. The K-means algorithm is applied iteratively, along with the calculations of the average co-membership matrices and similarity measures of cluster sets. When performing Tight Clustering on the class votes for a pair of pathways, the Euclidean distance between them is used. Tight Clustering does not explicitly estimate the number of clusters, but allows the user to specify the target number of Tight Clusters. It is usually infeasible to estimate the number of clusters since it is not uncommon to see figures that give clear and informative pattern for different number of clusters. For more details of the algorithm, see [9]. As mentioned in their paper, because microarray analysis is an exploratory tool to guide further biological investigations which could potentially be costly, some genes, called scattered genes in their paper, should be left out of the Tight Clusters. This is also true in the pathway-based context. Tight Clusters of class votes for pathways are found and pathways that are not related to other pathways should be left without being clustered. We varied the target number of Tight Clusters from 5 to 20 in analyzing the breast cancer data sets. Tight Clusters which contain two or more top ranked pathways with low OOB error rates are investigated further and pathway clusters are built from them. A heatmap can be used to visualize the Tight Clustering output.

**Figure 1:**
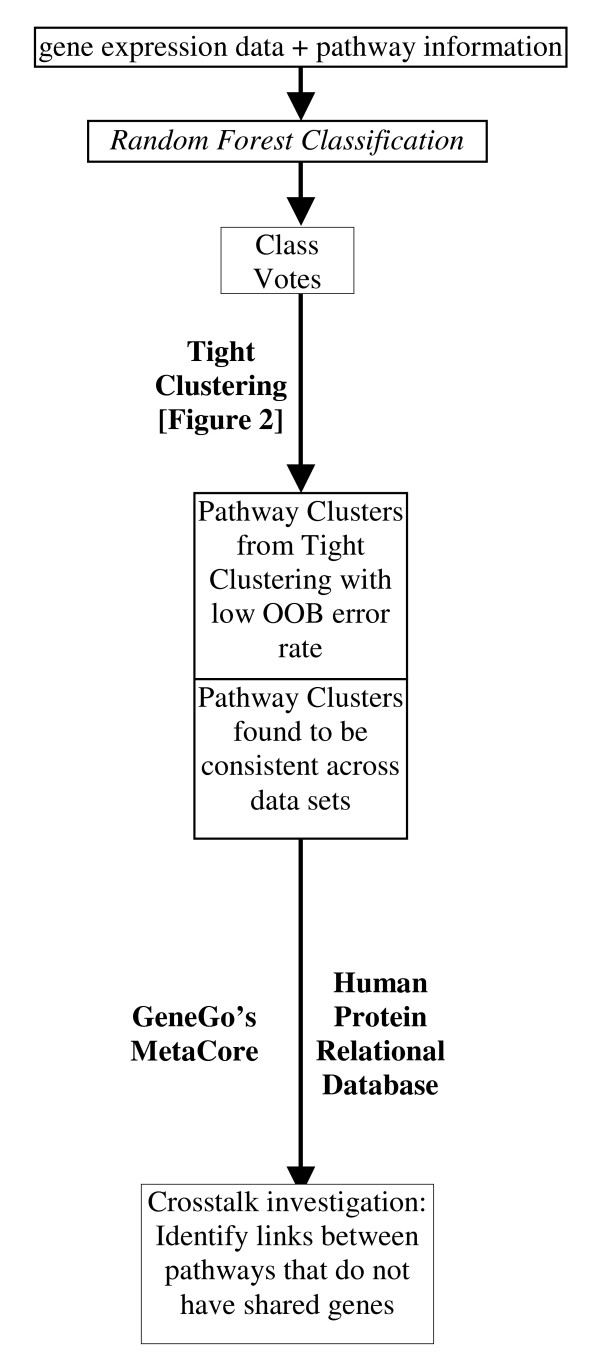
**A Schematic Diagram of How to Identify Clusters of Pathways**. Pathway (gene sets) information from externally available database, such as KEGG, BioCarta and GenMapp is combined with gene expression from clinical studies. We perform pathway-based Random Forests classification to obtain Class Votes. We identify clusters of pathways containing pathways with low OOB error rate using Tight Clustering. We identify the clusters of pathways that are consistent among different data sets. These pathway clusters are investigated further for possible crosstalk among them.

**Figure 2:**
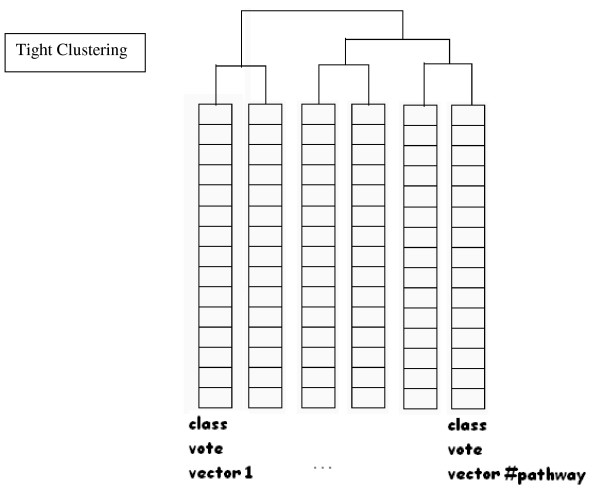
**Tight Clustering**. A diagram illustrating Tight Clustering on Class votes.

Schematic diagrams of our proposed methods are given in Figures 1 and 2.

### Data sets

#### Pathways

A total of 495 pathways were used for the analysis. These pathways are wired diagrams of a set of predefined genes and molecules from KEGG [1], BioCarta [2], and GenMapp [3] databases. Every pathway in these databases contains a set of genes that are related to some cellular, molecular and/or physiological functions from earlier experiments. These genes are then mapped to the corresponding probes IDs on the microarray chipsets. The distribution is as follows:

(1) A total of 151 pathways were taken from KEGG, a pathway database with the majority responsible for metabolism, degradation and biosynthesis. There are also a few signal or information processing pathways and others related to human diseases and drug development.

(2) We considered 283 BioCarta pathways. Most of these pathways are related to signal transduction for human with a smaller group of metabolic pathways.

(3) The 61 GenMapp pathways we used consist of more genes per set on average. There are different types of pathways such as metabolic pathways, signal transduction pathways, gene families and subcellular components.

#### Microarray data

Three different breast cancer microarray data sets were used. All of these studies used Affymetrix GeneChip^®^, but they are of different versions. *Consort *data set was based on hgu-133 plus 2.0 with 54,613 probesets whereas the other two, *LymphNode *and *p53 *data sets [10,11], were based on an older chip called hgu-133a with 22,215 probesets. *Consort *[12] data set consists of 99 breast tissue samples with clinical status of estrogen receptor. *LymphNode *data set consists of frozen tumor samples of 286 lymph-node negative patients who had not received adjuvant systematic treatment [10]. *p53 *data set is a set of 251 frozen tissues that were sequenced for p53 [11].

We chose the breast cancer data sets and ER positive/negative status (ER+/ER-) to study because breast cancer has been extensively studied in the literature and tumor samples are normally classified on the basis of ER status [13]. A recent publication described a set of prognostic gene expression classifiers for ER+ breast cancer [14]. The estrogen receptor status has also been used to predict breast cancer therapy, breast cancer survival rate and estimate the risk of breast cancer [15-18]. ER+ breast cancers are usually treated with hormone therapy whereas ER- breast cancers are treated using chemotherapy. Not all breast carcinomas are responsive to the treatment though. Thus, there is an urgent need to identify novel therapeutic targets and develop new agents. Moreover, pathway crosstalk and new biological insights might help find predictive biomarkers [19].

To deal with the issue of unbalanced sample size between the ER+ and ER- groups we utilized weighted Random Forests. The *p53 *data set is the most unbalanced among the three breast cancer data sets we analyzed, it has 213 in the ER+ and 38 in the ER- groups. For more details on why we chose this approach, see the discussion in the see Additional file 1, DMS1.

The above data sets are available for download from the GEO website under the accessions GSE2109, GSE3494 and GSE2034 for the *Consort*, *p53 *and *LymphNode *data sets, respectively. See Table 1.

**Table 1:** Breast cancer data sets used in this study

Data sets	Reference	n	Genes	Response type
*Consort*	INTEGEN	99	54613	ER status
*LymphNode*	[10] Wang (2005)	286	22215	ER status
*p*53	[11] Miller (2005)	251	22215	ER status

#### Software

The library package randomForest v4.5-18 from the R program was used in our analysis [20] for the Balanced Random Forests solution. A modified version of the original Fortran code was used to perform the Weighted Random Forests in our pathway-based context [21]. For pathway clusters visualization, Cytoscape [22] was used.

#### Biological Significance

We considered using GO terms based enrichment analysis, but Goeman and Bühlmann [23] pointed out that this approach may not be satisfactory and may result in false positives. Therefore, we used two alternative approaches. First, we used PubMatrix [24], a web-based application that identifies genes' citation with keywords of interest. Genes that contribute most in predicting the correct class in pathway-based classification are called informative genes [5]. We compared the informative genes defined by Random Forests classification that were obtained in the pathway cluster sets and examined whether these genes are more likely to have publications with the keywords of interest compared with informative genes from the top pathways not in the pathway cluster. Although importance measure in Random Forests could be biased [25], it is unlikely in our case since we only used normalized gene expression data and did not combine it with other categorical data, such as sequence data described in [25]. Second, we investigated possible pathways crosstalk using GeneGo's MetaCore [26] and Human Protein Reference Database (HPRD) [27]. Shortest path analyses between a pair of genes were performed using GeneGo's MetaCore to assess how close the two genes are related to each other based on the curated database of human protein-protein, protein-DNA and protein compound interactions.

## Results

### Class Votes

The target number of Tight Clusters, 5, 10, 15 and 20 were chosen and the tuning parameters were as defined in the Tight Clustering manual. We found that the pathway clusters identified when the target number was 5, 10 and 15 were essentially a subset of those in the 20 Tight Clusters case. To facilitate the investigation of pathway crosstalk, we consider a larger number of Tight Clusters, i.e. 20. We considered forming 25, 30, 35 tight clusters in addition to 5, 10, 15, and 20. Most of the clusters discovered in 20 tight clusters run were rediscovered in 25, 30, and 35. Please see Additional files 2 and 3, varysize_5-10-15-20.xls and varysize_20-25-30-35.xls for more details. On page 12 of the manual for the Tight Clustering program, four sets of parameters for tight clusters of size 5, 10, 15 and 20 were suggested. Therefore, we chose 20 tight clusters. Among these 20 inferred Tight Clusters, we selected those clusters containing two or more pathways whose OOB error rates were among the top 22 lowest across all the pathways. Since we aim to pick out the top pathways with the same OOB error rates, if we had chosen the top 20 pathways, we would have missed some pathways with the same OOB error rates. Based on this criterion, the OOB error rates cut off was 15.5%, 15.5%, and 20% for the *p53*, *LymphNode *and *Consort *data sets respectively. In each of the three data sets, three Tight Clusters were selected. These Tight Clusters are listed in Additional file 1, Table A1 for *Consort*; Table A2 for *LymphNode*; and Table A3 for *p53 *data set. A2ii and A3i from Additional file 1 for *LymphNode *and *p53 *data sets, respectively, highly resemble each other (Table 2). Apart from the Alzheimer's disease pathway, the other five pathways are overlapped between the *LymphNode *and *p53 *data sets. A2iii, A3ii and A1i in the respective data sets are also very similar (Table 3). "Butanoate metabolism", "Propanoate metabolism" and "Valine leucine and isoleucine degradation" appear in each of the three Tight Clusters of the three different data sets. The A3iii Tight Cluster in *p53 *data set is a subset of a much larger Tight Cluster A2i in *LymphNode*, see Additional file 1, Tables A2 and A3 for more details.

**Table 2:** Tight Cluster Results 1

*LymphNode *(A2ii in Additional file 1)	*OOB error(%)*	*Number of probes*
**BC-Pelp1_Modulation_of_Estrogen_Receptor**	**15.38**	**20**
Alzheimer's_disease	17.13	23
**BC-Deregulation_of_CDK5_in_Alzheimers_Disease**	**16.08**	**24**
**BC-Downregulated_of_MTA_3_in_ER_negative_Breast_Tumors**	**12.24**	**26**
**BC-GATA3_participate_in_activating_the_Th2_cytokine**	**11.89**	**33**
**Nitrogen_metabolism**	**14.34**	**40**
*Gene Symbols of informative genes in this pathway cluster*		
MAPK3, PELP1, ESR1, PDZK1, HSPB1, CA12, GLS, IL5, JUNB, GATA3, MAP2K3, MAPT, STH, CSNK1A1		

*p*53 (A3i in Additional file 1)		
**BC-Pelp1_Modulation_of_Estrogen_Receptor**	**13.94**	**20**
**BC-Downregulated_of_MTA_3_in_ER_negative_Breast_Tumors_**	**15.54**	**26**
**BC-GATA3_participate_in_activating_the_Th2_cytokine**	**13.55**	**33**
Nitrogen_metabolism	17.13	40
**BC-CARM1_and_Regulation_of_the_Estrogen_Receptor**	**14.34**	**54**
Gene Symbols of informative genes in this pathway cluster		
MAPK3, PELP1, ESR1, PDZK1, HSPB1, HDAC2, CA12, GLS, IL5, JUNB, GATA3, MAP2K3		

The bold pathways are those with low OOB error rates

**Table 3:** Tight Cluster Results 2

*LymphNode *(A2iii in Additional file 1)	*OOB error(%)*	*Number of probes*
**beta_Alanine_metabolism**	**16.08**	**42**
Alanine_and_aspartate_metabolism	16.78	42
**Glutamate_metabolism**	**16.08**	**50**
**Butanoate_metabolism**	**16.08**	**59**
Propanoate_metabolism	17.83	59
**Valine_leucine_and_isoleucine_degradation**	**14.69**	**71**
*Gene Symbols of informative genes in this pathway cluster*		
ABAT, ALDH1A3, GLUL, GMPS, HMGCL, HSD17B4, MAP3K15, MCCC2, PDHA1		

*p*53 (A3ii in Additional file 1)		
Alanine_and_aspartate_metabolism	17.53	42
**Butanoate_metabolism**	**15.54**	**59**
Propanoate_metabolism	18.33	59
GM-Glycolysis_and_Gluconeogenesis	23.11	66
**Valine_leucine_and_isoleucine_degradation**	**15.54**	**71**
*Gene Symbols of informative genes in this pathway cluster*		
ABAT, ALDH1A3, HMGCL, HSD17B4, MAP3K15, MCCC2, PDHA1		

*Consort *(A1i in Additional file 1)		
Glycosphingolipid_biosynthesis	23.23	34
**BC-GATA3_participate_in_activating_the_Th2_cytokine**	**14.14**	**43**
**Butanoate_metabolism**	**15.15**	**82**
Propanoate_metabolism	21.21	85
**Valine_leucine_and_isoleucine_degradation**	**17.17**	**25**
*Gene Symbols of informative genes in this pathway cluster*		
ABAT, GATA3, HSD17B4, MCCC2, PRKAR1B		

The bold pathways are those with low OOB error rates

### Pathway Clusters

**Figure 3:**
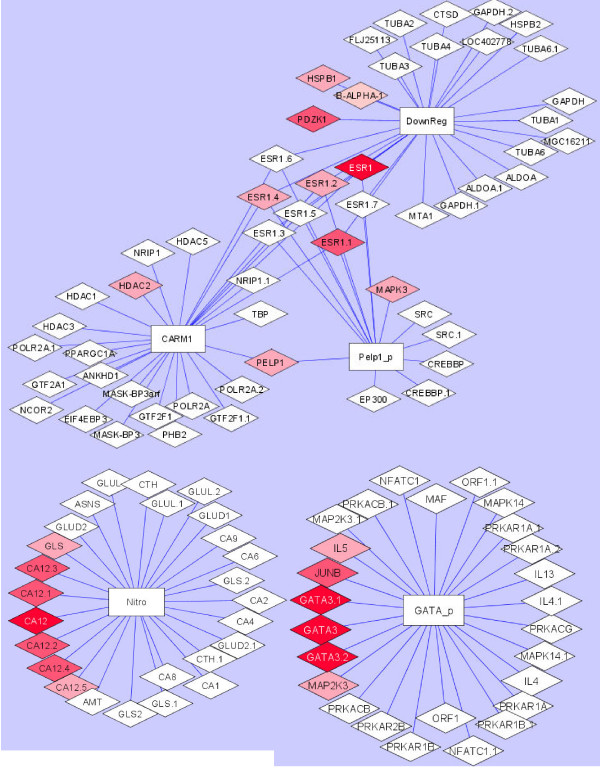
**Pathway Clusters**. A pathway cluster showing a total of five pathways, three of which have shared genes and two pathways do not share common genes.

We further investigate the pathway cluster (Table 2) found from the previous section. Figure 3 consists of three pathway clusters built from the overlapped pathways. It can be seen that "GATA3 participates in activating the Th2 cytokine gene pathway" and "Nitrogen Metabolism pathway" do not have any overlapping probes with the other 3 pathways. The ESR1 gene is shared among 3 pathways: "PELP1 Modulation of Estrogen Receptor Activity pathway", "CARM1 and Regulation of the Estrogen Receptor pathway", and "Downregulated of MTA 3 in ER negative Breast Tumors pathway". In addition to ESR1, the PELP1 and CARM1 pathways share the informative PELP1 gene. Genes, such as RARA, PGR, PDZK1, HSPB1, HDAC2, and MAPK3 that are not shared also show some importance in classifying subjects.

### PubMatrix

To more systematically study the biological significance of the results, we looked at the publications of the top informative genes (top two genes in each pathway) with keywords, like breast cancer, estrogen receptor, and progesterone receptor, of interest. We examined the top informative genes from the pathway cluster in Table 2, which consists of 5 pathways, with two of them that do not have any overlapping probes with the rest. It is evident from PubMatrix search that the proportions of these informative genes in the pathway cluster do show a higher number of literature support compared with the informative genes outside of the pathway cluster (Table 4). This is true for the informative genes for all three data sets and more so for the *p53 *data set. To assess the significance of these results, Fisher's Exact Test was performed. For breast cancer citations, the p-values were 0.149, 0.002, and 0.061 for data sets, *LymphNode*, *p53*, and *Consort*, respectively (Table 5). This indicates a significantly higher proportion of citations related to breast cancer for genes in pathway cluster of Table 2 compared to other informative genes in the top pathways for the *p53 *data set. The result for *Consort *just misses the significant cutoff of 0.05, and it is not significant for the *LymphNode *data set. It was not surprising to see more significant results for estrogen receptor citations, since we are specifically doing classification on the ER+/ER- status. All of the p-values are significant; 0.048, 0.0006, and 0.0084 for data sets *LymphNode*, *p53*, and *Consort*, respectively (Table 6).

**Table 4:** Proportion of genes showing more than the indicated number of literature support

Informative genes not in pathway cluster (Table 2) of top 22 pathways for *LymphNode*	BC	ER	PR
≥ 1	0.44	0.33	0.20
≥ 2	0.31	0.27	0.18
≥ 5	0.24	0.20	0.09

*LymphNode *(pathway cluster)	BC	ER	PR
≥ 1	0.42	0.42	0.33
≥ 2	0.42	0.42	0.25
≥ 5	0.25	0.33	0.17

Informative genes not in pathway cluster (Table 2) of top 22 pathways for *p*53	BC	ER	PR
≥ 1	0.41	0.35	0.20
≥ 2	0.33	0.28	0.13
≥ 5	0.24	0.24	0.11

*p*53 (pathway cluster)	BC	ER	PR
≥ 1	1.00	1.00	0.75
≥ 2	1.00	0.75	0.63
≥ 5	0.63	0.63	0.25

Informative genes not in pathway cluster (Table 2) of top 22 pathways for *Consort*	BC	ER	PR
≥ 1	0.50	0.22	0.16
≥ 2	0.44	0.38	0.25
≥ 5	0.34	0.28	0.13

*Consort *(pathway cluster)	BC	ER	PR
≥ 1	0.88	0.75	0.63
≥ 2	0.75	0.63	0.50
≥ 5	0.50	0.50	0.25

BC = breast cancer, ER = estrogen receptor, PR = progesterone receptor

**Table 5:** Breast Cancer Citations

*LymphNode*	In citation	Not in citation	p-value
Genes in pathway cluster (Table 2)	8	4	
Informative genes not in pathway cluster (Table 2) of top 22 pathways	20	25	0.149

*p*53	In citation	Not in citation	p-value
Genes in pathway cluster (Table 2)	8	0	
Informative genes not in pathway cluster (Table 2) of top 22 pathways	19	27	0.002

*Consort*	In citation	Not in citation	p-value
Genes in pathway cluster (Table 2)	7	1	
Informative genes not in pathway cluster (Table 2) of top 22 pathways	16	16	0.061

**Table 6:** Estrogen Receptor Citations

*LymphNode*	In citation	Not in citation	p-value
Genes in pathway cluster (Table 2)	8	4	
Informative genes not in pathway cluster (Table 2) of top 22 pathways	16	30	0.048

*p*53	In citation	Not in citation	p-value
Genes in pathway cluster (Table 2)	8	0	
Informative genes not in pathway cluster (Table 2) of top 22 pathways	15	30	0.0006

*Consort*	In citation	Not in citation	p-value
Genes in pathway cluster (Table 2)	6	2	
Informative genes not in pathway cluster (Table 2) of top 22 pathways	7	25	0.0084

### Possible Pathway Crosstalk

**Figure 4:**
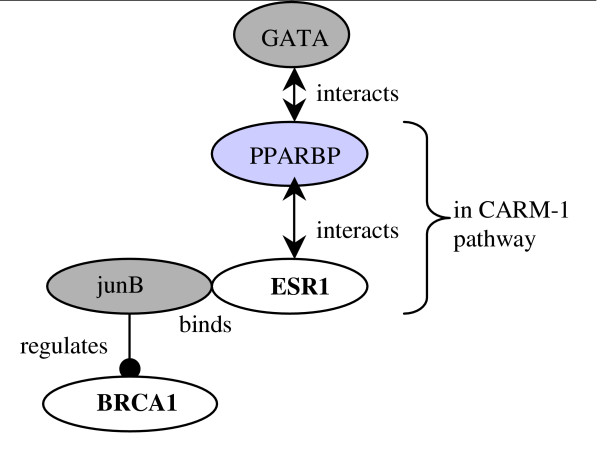
**Links between GATA3 and CARM1 pathways using HPRD**. The connection between genes in GATA3 and CARM1 pathways using information obtained from HPRD.

From the previous section, we have seen that even though there are no overlapping genes between both "Nitrogen metabolism" and "GATA3 participate in activating Th2 cytokine genes expression" pathways with other pathways containing ESR1, they appear to form a tight pathway cluster. In order to further understand the possible crosstalk between them, we looked at HPRD and GeneGo's MetaCore. We found connections between GATA3 pathway and CARM-1 pathway from HPRD. This is illustrated in Figure 4, where the dark grey oval genes GATA and junB in GATA3 pathway interacts with PPARBP and ESR1 in CARM-1 pathway. The gene PPARBP, Peroxisome proliferator-activated receptor binding protein, is determined to be at a high level of expression and amplified in breast cancer [28]. In Figure A5 in Additional file 1, it suggests how different proteins receive signals from ESR1 and act upon HIF-1 to regulate CA12.

### Shortest Path Analyses

To investigate the possibility of pathway crosstalk further, we searched for the shortest path between GATA3 and CA12 with other top informative genes in the network of all links in the database of GeneGo's MetaCore. This tool assists in finding regulatory paths between two or more genes of interest. The results are shown in Tables 7 and 8. For both GATA3 and CA12, they are the genes with the least number of gene steps to the gene ESR1, with 2 and 3 steps, respectively. It furthers strengthens our belief that the pathways GATA3 and Nitrogen Metabolism are closely tied with the other four pathways within the pathway cluster. The number of links with a distance of two between GATA3 and ESR1 is 6, which is much larger than ESR1 and EGFR or IKBKB both with just one gene connecting between them. The gene, MUC1, connects ESR1 and EGFR. IKK-alpha is the gene which connects ESR1 and IKBKB. These two genes are a subset of the 6 between GATA3 and ESR1. Furthermore, there are 7 literature support of genes MUC1 and HNF3-alpha [29-35] related to breast neoplasm compared to 6 (MUC1) for EGFR and none for IKBKB. In fact, EGFR is one of the genes in the Calcium Signalling pathway which also share genes with the GATA3 pathway. CA12 and ESR1 are also closely tied; CA12 is connected to ESR1 through HIF-1 and NCOA1. There are 4 literature support of genes HIF-1 and NCOA1 related to breast neoplasm [36-39]. Again, the EGFR is at the top of this chart with the same number gene steps but with 4 different paths, and two more literature support than CA12. Another gene IL6ST has two different paths and three literature support. Although, it seems that the connection between CA12 and the four pathways with ESR1 is not as strong, it is still significant relative to the majority of the top informative genes which show 4 or more gene steps.

**Table 7:** Shortest Path between GATA3 and other Genes in the Top 22 Pathways (without overlap with GATA3 pathway)

**GATA3 and**	**Distance (gene steps)**	Number of links with the shortest distance*	Genes with literature related to breast cancer*
**ESR1 **(in pathway cluster)	2	6 [MUC1, SMAD8, IKK-alpha, HDAC4, HNF3-alpha, GATA-1]	7
**EGFR**	2	1 [MUC1]	6 (subset of the 7 above)
**IKBKB**	2	1 [IKK-alpha]	0
**IFNAR**	3		
**GFRA1**	3		
**IGF1**	3		
**ATP7B**	3		
**VAV3**	3		
**COX7c**	3		
**B3GNT6**	3		
**MYCL1**	3		
**ACACB**	3		
**UQCRH**	3		
**LYN**	3		
**ACTN1**	3		
**IL6ST**	3		
**STC2**	4		
**PDXK**	4		
**CFLAR**	4		
**BBOX1**	4		
**TARS**	5		
**SSH3**	5		
**NDUFA9**	6		
**HMGCL**	6		
**ABAT**	6		
**DAZAP2**	Infinity		

*shown only for the shortest distance

**Table 8:** Shortest Path between CA12 and other Genes in the Top 22 Pathways (without overlap with Nitrogen Metabolism pathway)

**CA12 and**	**Distance (gene steps)**	Number of links with the shortest distance*	Genes with literature related to breast cancer*
**ESR1 **(in pathway cluster)	3	1 (HIF-1, NCOA1)	4
**EGFR**	3	4 (HIF-1 + MAPK3, Beta-catenin, STAT5B, MAPK1)	6
**IL6ST**	3	2 (HIF-1 + MAPK1, MPAK3)	3
**COX7c**	4		
**IGF1R**	4		
**YES**	4		
**ACTIN1**	5		
**PRKX**	5		
**VAV3**	5		
**ABAT**	6		
**HMGCL**	6		
**SSH3**	6		
**TARS**	6		
**ADCY9**	7		
**BBOX1**	7		
**PDXK**	7		
**UQCRH**	7		
**B3GNT6**	9		
**DAZAP2**	Infinity		

*shown only for the shortest distance

## Discussions

In this article, we have described a Random Forests-based approach to identify clusters of pathways sharing similar functions. Class votes measure similarity at the individual level. Using the three different breast cancer data sets to classify between estrogen receptor positive and negative status, we found that Tight Clustering for class votes yielded consistent and interpretable results. We also considered other means of measuring the similarity of class votes, such as the similarities between class votes solely by Euclidean distances, but their performance was less consistent than the methods described here. Moreover, another output, proximity matrices, for Random Forests was also investigated, but it was found to be highly correlated with the class votes (see Figures A4.). Bioinformaticians and biologists can make use of the proposed methods to discover pathway clusters, find informative genes shared between pathways and identify genes that bridge between pathways within a pathway cluster. This allows researchers to obtain results that are more closely tied to the biological mechanism of diseases and to examine pathway crosstalk.

Due to the unbalanced nature of the data sets in this study, the weighted random forests (WRF) algorithm was used. WRF seems to perform better than the alternative balanced random forests procedure. Although we are looking at ER+ vs. ER- status for the *Consort*, *p53 *and *LymphNode *data sets, it is reasonable to obtain different pathway clusters from them. This is because the patients were from different clinical settings. The *Consort *data set consists of patients from a consortium of different breast cancer studies, the *p53 *data set consists of patients whose tissue were sequenced for *p53 *and the *LymphNode *data set only has patients with negative lymph node status.

In this article, we have also demonstrated the biological relevance of our approach using PubMatrix. The number of citations for informative genes within the pathway cluster together with keywords, like estrogen receptor, is enriched compared to other informative genes of top pathways. We have illustrated the use of GeneGo and HPRD to help us understand possible crosstalk among pathway clusters. The shortest path analyses of GATA3 and CA12 show that the informative genes in pathway clusters are closer in terms of regulatory paths than those informative genes in other top pathways. Furthermore, with the aid of a network visualization tool, biologists can investigate how the informative genes are related to each other within the pathway clusters.

## Conclusion

The novel methods presented in this article were able to identify pathway clusters related to outcome of interests that are biologically meaningful. Understanding how the informative genes relate and talk with each other within pathway clusters can help generate further biological hypotheses for follow-up studies. These may be tested using other "omics" technologies, such as proteomics and metabolomics. When the outcome variable is continuous, we can employ the Random Forests Regression approach [5] and easily extend what we have described in this article to the regression setting by using the predicted values from the Random Forests output.

In this paper, we have proposed one way to building pathway clusters. It might be possible to utilize output from other pathway-based methods, such as GSEA to determine the similarity in enrichment scores between two pathways and build a graph of pathway network from the calculated similarity measures. Moreover, our approach would encourage other researchers to look into new ways in building pathway clusters and bring fresh insights into microarray analysis.

## Authors' contributions

HP and HZ developed this new method for building pathway clusters. HP did the programming and carried out the computational work. Both authors read and approved the manuscript.

## Supplementary Material

Additional file 1 This file contains supporting information for this paper. These include: a study of weighted random forests vs. balanced random forests, GeneGo MetaCore output and tight clusters and absolute differences results. Click here for file

Additional file 2 Tight Clustering results for 5, 10, 15 and 20 tight clusters. Click here for file

Additional file 3 Tight Clustering results for 20, 25, 30 and 35 tight clusters. Click here for file
