# Supplementary material for: Building pathway clusters from Random Forests classification using class votes
Source: BMC Bioinformatics. 2008 Feb 6;9:87. doi: 10.1186/1471-2105-9-87 (PMC2335306; doi:10.1186/1471-2105-9-87)
Supplement: Additional file 1 — This file contains supporting information for this paper. These include: a study of weighted random forests vs. balanced random forests, GeneGo MetaCore output and tight clusters and absolute differences results. [file 1471-2105-9-87-S1.DOC]

**Supporting Information for:**

Building pathway clusters from Random Forests Classification using class votes

Herbert Pang^1^ and Hongyu Zhao^1,2^

^1^Division of Biostatistics, Department of Epidemiology and Public Health and ^2^Department of Genetics, Yale University School of Medicine, New Haven, CT, 06520 USA

Contents of Supporting Information

# Figures

A1. ROC curve for Consort data set 3

A2. ROC curve for LymphNode data set 3

A3. ROC curve for p53 data set 4

A4. Correlation between class votes and proximity matrices 5

A5. GeneGo MetaCore results - How CA12 is related to ESR1 7

# Tables

A1. Tight Clusters (Consort) 1

A2. Tight Clusters (LymphNode) 1

A3. Tight Clusters (p53) 1

A4. Comparison of performance metrics for BRF and WRF (Consort) 2

A5. Comparison of performance metrics for BRF and WRF (LymphNode) 3

A6. Comparison of performance metrics for BRF and WRF (p53) 3

# Others

DMS1. Unbalanced Data sets 2

**Table A. Tight Clusters**

**Cluster Votes**

***Table A1: Tight Clusters (Consort)***

***Table A2: Tight Clusters (LymphNode)***

***Table A3: Tight Clusters (p53)***

## DMS1. Unbalanced data sets

Many breast cancer tumors are hormone-dependent and have receptors for estrogen and/or progesterone. Thus, the majority of patients are ER positive. We have to deal with the imbalanced data issue in Random Forests which was addressed earlier (Chen et al. 2004). We can use one of the two schemes of Random Forests: Balanced Random Forests (BRF) and Weighted Random Forests (WRF). The BRF draws for each run of a random forest a bootstrap sample from the minority class and randomly draw a sample with replacement of the same number from the majority class. This is then repeated a number of times and the results are aggregated just like the original Random Forests algorithm. The difference between the WRF and the ordinary Random Forests is that class weights are used to compute the weighted Gini criterion for doing splits. At the terminal node, weighted votes are taken to determine the prediction for the node. The weighted vote of a class is defined as the class weight multiplied by the number of cases of a particular class at the terminal node.

Given the confusion matrix from Random Forests classification

|  | True Class | |
| --- | --- | --- |
|  | Positive | Negative |
| Predicted Positive | TP (True Positive) | FP (False Positive) |
| Predicted Negative | FN (False Negative) | TN (True Negative) |

The performance metrics used include: True Negative Rate (TNR) = TN/(TN+FP), True Positive Rate (TPR) = TP/(TP+FN), G-mean = √(TNR×TPR), Weighted Accuracy = 0.5(TNR + TPR), Precision = TP/(TP+FP), F-measure = (2×Precision×Recall)/(Precision + Recall). For more details regarding these measures, see Chen et al. (2004).

We compared BRF and WRF to see which one works better for the three different data sets we have. For BRF, the cutoff for votes for the final prediction can be changed and by varying this we can get a range of True Positive Rates and False Positive Rates. Similarly for WRF, when the weights vary we can also get a set of the different rates.

The top overlapping pathways across the different cutoffs and weighting schemes for the BRF and WRF methods respectively were chosen to obtain the performance metrics for comparison. The top 8 pathways were chosen and we take an average of these measures.

From Figures A1-A3, we see that for the consort cancer data, the ROC curves for both BRF and WRF are very similar. However, for the other data sets, WRF outperforms BRF. We can see a similar pattern for the different performance metrics such as the F-measure, G-mean and Weighted Accuracy, please see table A1-A3. The cutoff was chosen according to the ROC curve.

# Table A4

| Consort | TPR | TNR | Precision | F-measure | G-mean | Wt. Accuracy |
| --- | --- | --- | --- | --- | --- | --- |
| wrf=1:1.5 | 81.79% | 84.30% | 74.90% | 78.13% | 83.00% | 83.05% |
| brf=0.4 | 90.63% | 77.78% | 69.98% | **78.94%** | **83.93%** | **84.20%** |
| brf=0.5 | 82.99% | 84.13% | 75.00% | 78.75% | 83.53% | 83.56% |

# Table A5

| LymphNode | TPR | TNR | Precision | F-measure | G-mean | Wt. Accuracy |
| --- | --- | --- | --- | --- | --- | --- |
| wrf=1:2.5 | 85.55% | 85.94% | 69.23% | **76.51%** | **85.74%** | **85.75%** |
| brf=0.4 | 83.01% | 80.15% | 63.57% | 71.51% | 81.24% | 81.58% |
| brf=0.5 | 82.96% | 81.04% | 63.74% | 71.59% | 81.68% | 82.00% |

# Table A6

| p53 | TPR | TNR | Precision | F-measure | G-mean | Wt. Accuracy |
| --- | --- | --- | --- | --- | --- | --- |
| wrf=1:4 | 74.34% | 87.09% | 50.81% | **60.31%** | **80.44%** | **80.72%** |
| brf=0.4 | 72.70% | 73.18% | 32.86% | 45.20% | 72.92% | 72.94% |
| brf=0.5 | 57.89% | 87.85% | 47.38% | 51.62% | 71.19% | 72.87% |

Figure A1 – Consort data set

# Figure A2 – LymphNode data set

Figure A3 – p53 data set

From these performance metrics, we chose to use weighted Random Forests as our strategy to tackle the unbalanced data problem. In the paper by Chen et al. (2004), the authors noted that WRF may favor certain data sets than others. In determining the weight to be used, there is a tradeoff between true positive rate and false positive rate. The weights 1.5, 4 and 2.5 are used to put on the minority class, for the consort, p53 and LymphNode data sets respectively. These weight appear to be at the shoulder of the ROC curves and have highest F-measure, G-mean and weighted accuracy.

#

# Figures A4 Correlation between class votes and proximity matrices

# We have also investigated the possibility of using Proximity Matrices for the identification of pathway clusters. Apart from the class vote vector, Random Forests also provides an n × n proximity matrix to measure the similarity between subjects. To obtain this measure, both the training set and OOB data are run down a tree. If case 1 and case 2 both result in the same terminal node, then the proximity between case 1 and case 2 is increased by one. The normalized proximity measure is the total count divided by the number of trees. The proximity matrix allows us to measures the similarities between the individuals based on gene expression information from each pathway.

# We discovered that the two measures are highly correlated, please see the figures below:

Top 22 pathways for Euclidean Distances on Class Votes vs. Absolute Differences on Proximity Matrices (*Consort*)

# Top 22 pathways for Euclidean Distances on Class Votes vs. Absolute Differences on Proximity Matrices (*LymphNode*)

# Top 22 pathways for Euclidean Distances on Class Votes vs. Absolute Differences on Proximity Matrices (*p53*)

#
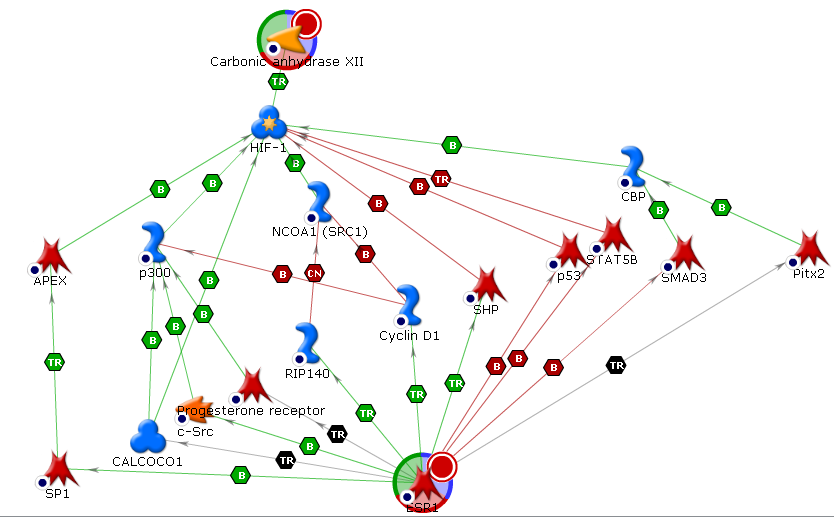


# Figure A5 - How CA12 is related to ESR1 from GeneGO

REFERENCES:

Chen, C., Liaw, A., Breiman, L. (2004). Using Random Forest to Learn Imbalanced Data. <http://oz.berkeley.edu/users/chenchao/666.pdf>

Pang, H., Lin, A., Holford, M., Enerson, B. E., Lu, B., & Lawton, M. P., et al. (2006). Pathway analysis using random forests classification and regression. Bioinformatics (Oxford, England), 22(16), 2028-2036.

<http://bioinformatics.oxfordjournals.org/cgi/content/full/22/16/2028>
